# Repeated Mirror Exposure in Individuals With Body Dysmorphic Symptoms

**DOI:** 10.32872/cpe.19097

**Published:** 2026-05-29

**Authors:** Katrin Schoenenberg, Alexandra Martin

**Affiliations:** 1 Department for Clinical Psychology and Psychotherapy, Kiel University, Kiel, Germany; 2 Department for Clinical Psychology and Psychotherapy, University of Wuppertal, Wuppertal, Germany; Philipps-University of Marburg, Marburg, Germany

**Keywords:** body dysmorphic disorder, body image exposure, body satisfaction, shame, post-event processing

## Abstract

**Background:**

Mirror exposure represents a common component in treatment of body dysmorphic disorder (BDD). However, the benefits of repeated mirror exposure have not been investigated as a standalone intervention for BDD. This study aimed to examine the cognitive and affective response to mirror exposure in individuals with high and low levels of body dysmorphic symptoms.

**Method:**

Fifty women participated in two guided full-body mirror exposures (approx. 32 minutes each). Participants were divided into two groups based on the severity of their BDD symptoms. Twenty-three participants reported elevated, not primarily weight related, body dysmorphic symptoms. Body satisfaction and affective responses were assessed before and after the exposure, affective responses were further assessed during the exposure. Post-event processing related to the experience was rated the day afterwards.

**Results:**

Participants with body dysmorphic symptoms reported lower state body satisfaction and higher shame in both sessions, sadness was elevated in the first session only. State body satisfaction dropped from pre to post exposure but improved from the first to the second session. Negative affects did not decrease within but between the two sessions. Post-event processing after the first exposure predicted negative affect at the beginning of the second session.

**Conclusion:**

The results support a positive effect of repeated mirror exposure across sessions, without improvement within the session. They point towards the detrimental role of mental post-processing.

## Highlights

- Examining the effect of repeated mirror exposure in BDD-symptomatic women.
- State body satisfaction improves between sessions, but is impaired post exposure.
- Women with BDD symptoms experienced more pronounced feelings of shame.
- Negative affects and post-event-processing improved across sessions.

Body Dysmorphic Disorder (BDD) is characterized by excessive preoccupation with one or more subjective flaws in appearance and associated distress or impairment in daily life (American Psychiatric Association, 2013). According to cognitive-behavioral BDD models (Veale & Neziroglu, 2010; Wilhelm et al., 2013) and experimental studies (Barnier & Collison, 2019; Kollei & Martin, 2014; Waldorf et al., 2019), looking into a mirror elicits dysfunctional processing of one’s own appearance. As in other body image disorders, mirror checking and mirror avoidance are common safety behaviors in BDD (Naumann et al., 2022). Mirror checking serves as a behavioral response to uncertainty about appearance and is motivated by the hope to be less dissatisfied with the appearance, at the same time it increases selective attention to the disliked body part(s) (Veale & Riley, 2001). Additionally, it is not uncommon for those affected to also avoid, either completely or partially, situations in which they are confronted with their own appearance and thus with the associated aversive feelings. This also strengthens dysfunctional processing of the self and thus selective attention (Veale & Neziroglu, 2010). Selective attention in BDD refers to threatening stimuli associated with rejection or judgment (Johnson et al., 2018), as well as disorder-related stimuli such as body parts.

Kollei and Martin (2014) found that individuals with BDD expressed more body-related and more negative cognitions than participants who were either in good health or suffering from a major depression when confronted with their bodies in the mirror. On the day after the exposure, participants with BDD reported more post-event processing than controls. The results on post-event processing were supported by Schoenenberg and Martin (2022) in people with elevated BDD concern. Neziroglu and colleagues (2010) investigated mirror gazing in BDD and healthy participants at one-minute intervals. The levels of fear and disgust were higher in the BDD group, but decreased significantly over time.

Satisfaction with one’s own body or appearance represents the cognitive-evaluative component of body image (Cash, 2012). In a study by Waldorf et al. (2019), men with the BDD subtype muscle dysmorphia had a generally lower level of state body satisfaction and higher negative affect scores than weight-trained or non-weight-trained controls. When confronted with images of their own body, participants in all the groups responded with a lower level of body satisfaction, but negative affect increased only in the muscle dysmorphia group. No study has investigated state body satisfaction in women with elevated BDD symptoms in the context of mirror exposure so far. For this reason, we aimed to assess state body satisfaction pre and post isolated mirror exposure. We hypothesized the following:

**Hypothesis I (BDD group effect)**: Individuals with distinct BDD symptoms express overall lower state body satisfaction (H1a) and stronger negative affect (H1b) in the context of mirror exposure than individuals with low or no BDD symptoms. Furthermore, individuals with distinct BDD symptoms experience more post-event processing (H1c) about the exposure the following day than a group with low or no BDD symptoms.

In the treatment of BDD, mirror exposure serves to correct attentional and evaluative biases (Veale & Neziroglu, 2010; Wilhelm et al., 2013). Typically, individuals are asked to describe their body in, e.g., a neutral, or non-judgmental way when confronted with their body image in the mirror (Griffen et al., 2018; Schoenenberg & Martin, 2022). Compared to short mirror viewing, it is common to guide the attention to different body parts during mirror exposure to counteract poor global processing and to train finding a less judgmental way of describing the body. It takes longer than short viewing or checking and follows a different approach which is why some manuals prefer the term “mirror retraining” (Wilhelm et al., 2013). Even though mirror exposure or retraining is part of cognitive behavioral treatment manuals, no study has investigated its isolated effect on individuals with BDD (Griffen et al., 2018). Due to the experimental character of the present study the term “mirror exposure” will be used in the following even though we did apply a typical retaining rationale including guidance through the body parts, with instructions to observe upcoming thoughts and emotions and to use a neutral description of the perceived body parts.

Reviews of research on eating disorders summarized the positive effects of repeated video and mirror exposure (Butler & Heimberg, 2020). The few studies on body image exposure in samples with weight-related body dissatisfaction examining within and between sessions effects in detail tend to support between-session effects (Díaz-Ferrer et al., 2017; Moreno-Domínguez et al., 2012). To our knowledge, the effects of mirror exposure within and between sessions with respect to body image evaluations and affective responses in persons with distinct BDD symptoms have not yet been studied. Therefore, we tested the following hypotheses:

**Hypothesis II (within-session time effect)**: At the end of a mirror exposure session, state body satisfaction is higher (H2a) and negative affect is lower (H2b) in comparison to at the beginning of the session.

**Hypothesis III (between-session effect)**: In a second mirror exposure session, body satisfaction is higher (H3a); negative affect is lower (H3b) and post-event processing is lower (H3c) compared to in the first session.

As a third goal, this study aimed to uncover the potentially destructive role of post-event processing in maintaining the disorder. This mechanism is well-known and well-studied for social anxiety (e.g., Fehm et al., 2008; Rachman et al., 2000). It refers to a negatively biased way of reprocessing a previous difficult event and is considered a special form of rumination. Negative distorted post-processing could lead to change in the memorized experience and could interfere with the correction of one's own expectations. If post-event processing contributes to maintenance of appearance-related concerns, stronger post-event processing after a first mirror exposure should predict a poorer body image at the onset of the second session. This could also be of therapeutic relevance, as the barrier to so far avoided mirror exposure could be higher with more pronounced post-event processing and could possibly even risk dropout. Accordingly, we investigated the following hypothesis:

**Hypothesis IV (model prediction):** Post-event processing about the first mirror exposure predicts lower state body satisfaction (H4a) and higher negative affect (H4b) at the beginning of the second exposure.

To test these hypotheses, we invited individuals with body dysmorphic symptoms and a group without (or with only a low level) of BDD symptoms to participate in the study. All participants took part in two guided mirror exposure sessions separated by a one-week interval. The exposure procedure was standardized and similar to mirror exposure in BDD treatment. No additional intervention components were provided to the study participants.

## Method

### Design

The experimental study had a 2 (BDD *group*: positive, negative; between factor) x 2 to 4 (*time*: pre, full-body exposure beginning, full-body exposure end, post; within-factor) x 2 (*session*: first, second; within-factor) mixed design.

### Participants

Participants were included if they were female and between 18 and 55 years old. Based on a screening, participants who were allocated to the BDD positive group were additionally checked by asking them to name their primary area of concern. If weight was their primary area of concern, they were excluded from participation. Males were excluded from participation because the exposure in underwear to the female examiner could have been a confounding factor. Participants who reported suicidal tendencies were furthermore excluded from participation.

The fifty female participants were on average 22.8 years old (*SD* = 4.5). As the highest education level, 82% reported a high-school degree and 18% a university degree. Most participants were living in a relationship (50%) or were single (44%), and some were married (6%). The sample had a body mass index (BMI) in the normal range (*M* = 21.89, *SD* = 2.95). Participants with elevated BDD-symptoms were most dissatisfied with their nose (21.7%), belly (17.4%), legs (13.0%), teeth (8.7%) and hands (8.7%). Participants with little BDD-symptoms reported to be most dissatisfied with their belly (22.2%), breast (14.8%), nose (14.8%), body shape (7.4%), legs (7.4%) and teeth (7.4%). All other body parts were named only once in each of the two groups.

### Materials

#### Sample Characteristics and Manipulation Check

The participants indicated ***demographic questions*** regarding their age, gender, relationship status, and highest educational attainment.

The ***Dysmorphic Concern Questionnaire*** (DCQ; Oosthuizen et al., 1998) is a well-established screening instrument for BDD. Its seven items assess the extent of body dysmorphic concerns on 4-point Likert scales (sum range 0 to 21). Different studies have confirmed its good level of reliability and validity (e.g., Schieber et al., 2018; Stangier et al., 2003; α = 0.88 in the current study). In student samples, a cut-off value of nine has proven to be the best balance between sensitivity and specificity (Mancuso et al., 2010). For this reason, participants were assigned to the BDD positive *group* (BDD+; *N*_BDD+_ = 23) if they scored nine or higher and to the BDD negative *group* (BDD-; *N*_BDD-_ = 27) if they scored eight or lower.

To assess BDD criteria (DSM-5), a ***self-rated BDD diagnosis*** scale that had previously been used in epidemiologic studies was employed (Schieber et al., 2015). If all criteria were answered with ‘yes’ and if participants indicated spending at least one hour a day thinking about their appearance or taking actions related to their appearance, and primary weight concerns were excluded, the participants were considered to have a self-rated BDD diagnosis (*N* = 11). We also recorded the ***body part*** that participants were most dissatisfied with via a self-report question.

Further assessments calculated the appearance ***distortion*** by the difference between the examiners’ and the participants’ ratings on an 11-point scale from 0 “not at all affected in appearance” to 10 “very strongly affected in appearance” (Stangier et al., 2000). Verbal anchors on the scale supported the examiners’ assessment (0: not impaired in appearance at all, 2: slightly impaired in appearance, 5: impaired in appearance; 7: severely impaired in appearance, 10: very severely impaired in appearance).

The ***Patient Health Questionnaire Depression Module*** (PHQ-9; Kroenke et al., 2001) measures depression severity with nine items. The items scale ranges from 0 “not at all” to 3 “nearly every day.” Its internal consistency was excellent in the original validation study (α = 0.86 and 0.89; Kroenke et al., 2001) and acceptable in the present study (α = 0.78).

In a short, ***structured interview*** after the exposure, the ability and motivation to follow instructions during the exposure and the stress induced by the exposure were assessed to verify the intended manipulation using 11-point Likert scales from 0 “not at all” to 10 “very much” (Kollei & Martin, 2014; see Supplementary Materials, Table 1).

#### Dependent Variables

The ***Body Image State Scale*** (BISS; Cash et al., 2002) assesses body image satisfaction. The mean score of the six items (scale from 1 to 9) is typically used. The BISS showed an acceptable to good internal consistency in prior studies with women samples (α = 0.77 to 0.90; Cash et al., 2002; Vocks et al., 2009) as well as in this study (α = 0.79 to 0.89).

The intensity of the four ***negative affects*** tension, shame, sadness, and anxiety were assessed on visual analogue scales ranging from 0 “none” to 100 “strong”. The examiner verbally asked the subjects to rate their affect intensity before, during, and after exposure.

The ***Post-Event Questionnaire*** (PEP-Q; Fehm et al., 2008; Rachman et al., 2000) assesses repetitive negative thinking about a past difficult event. We used an adapted version for mirror exposure (Kollei & Martin, 2014) that was administered 24 hours after the exposure. The participants completed the questionnaire online via the LimeSurvey tool. Sixteen items (scale from 0 “none, never, not at all” to 100 “very strong, always”) addressed processing about mirror exposure in the past 24 hours. This version showed excellent internal consistency both in prior studies (α = 0.95, Kollei & Martin, 2014; α = 0.89 to 0.93, Schoenenberg & Martin, 2022) and in this study (α = 0.95).

### Procedure

The ethics committee of the University of Wuppertal approved the study (MS/BBL 190326 Schoenenberg). Participants were recruited via flyers, online postings, and mailing lists for a study on repeated mirror viewing, with no indications in the advertisement about potentially beneficial effects of the intervention. The participants received detailed information about the study procedure, data protection, and their rights as participants prior to inclusion in the study, and provided informed consent.

**Figure 1:**
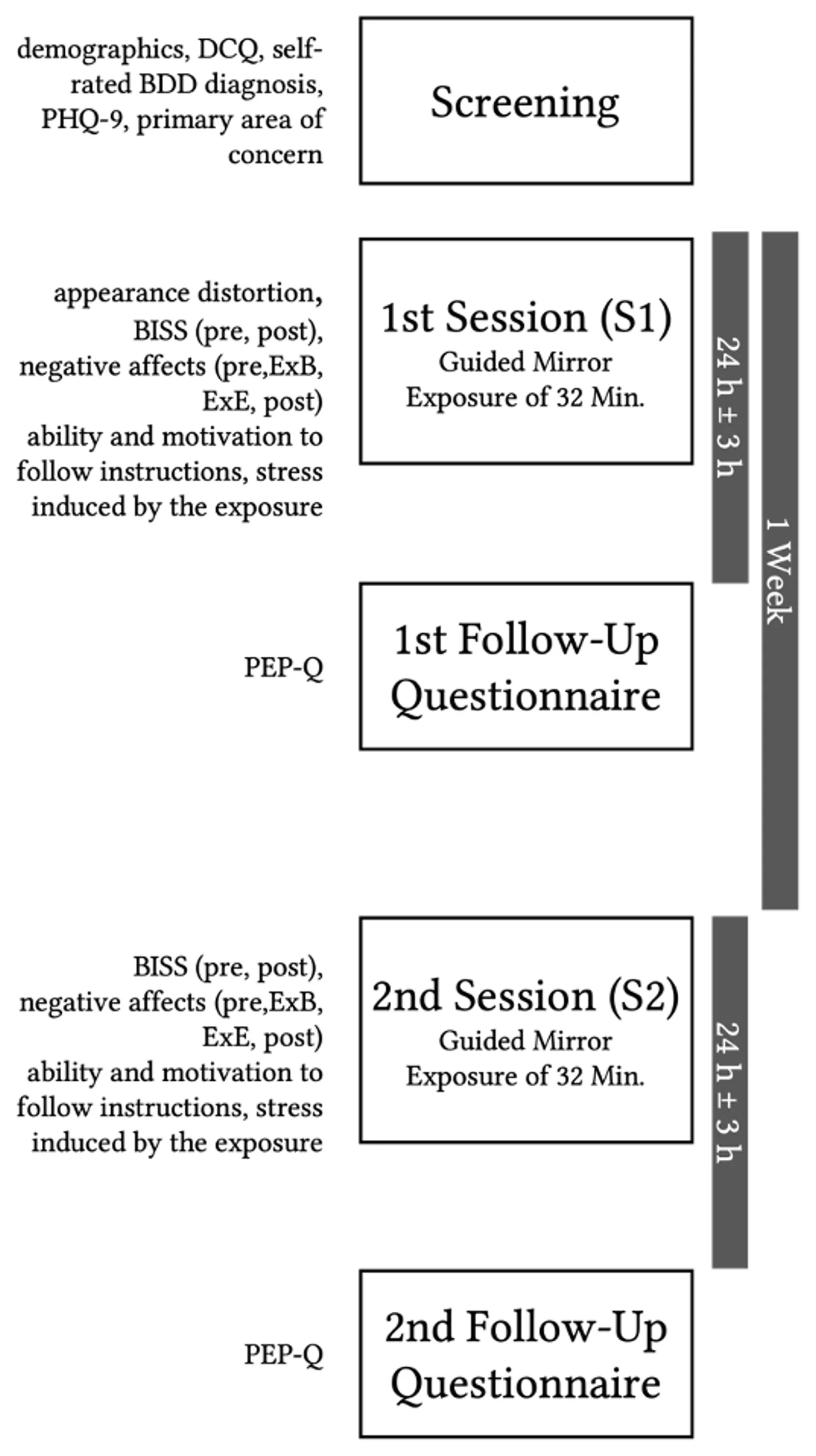
Procedure of Repeated Mirror Exposure Study *Note.* DCQ = Dysmorphic Concern Questionnaire; PHQ-9 = Patient Health Questionnaire Depression Module; BISS = Body Image State Scale; PEP-Q = Post-Event Questionnaire; Pre = pre-exposure; ExB = full-body view beginning; ExE = full-body view end; Post = post-exposure.

Prior to the laboratory appointment, participants responded to an online screening questionnaire (Figure 1) that checked the inclusion and exclusion criteria. Those eligible to participate were invited to attend a first mirror exposure session in the laboratory of the University of Wuppertal. Here, they were again informed about the procedure, and they then responded to a first questionnaire including the first rating of state body satisfaction. The examiner introduced the participant to the task during the mirror exposure, which involved describing all named body parts in detail and in a neutral way. Experience of negative affect and cognitions were permitted and registered but the focus was on describing features of the body or body part in a neutral manner.

Sample questions were given to participants, e.g., “What is the color/size/texture of this body part?”. A sample text with a description of eyes was provided, and participants were asked to describe their thumb to practice the procedure. Afterwards, they undressed to their underwear, which was meant to have a neutral color and design. We chose underwear as clothing to ensure participants could hide almost none of their potential flaws with their clothes. Psychophysiological measurement equipment for assessing respiration, heart rate, and electrodermal activity was attached to their back with a belt.

Before exposure, the participants were asked to position themselves at a mark 50 cm away from a full-body mirror (height: 175 cm; width: 69 cm; adjustable wings) with their backs to the mirror for two minutes. They were then asked to provide their first affect rating (pre). Afterwards an audio recording with instructions guiding participants through the exposure was played. At first, the participants were asked to look at their entire body for one minute. This was followed by the second affect rating (ExB). The examiner left the room, and the recording directed the subjects’ attention to the different body parts of the head, upper and lower torso, and the back view of the body. The female voice prompted them to describe the respective body parts. The participants described their bodies internally to reduce shame and artefacts in psychophysiological measurement. The recording ended with a second full-body view from the front. The recording from the first full body view until this last full body view lasted approximately 32 minutes. The examiner entered the room and assessed affect for the third time (ExE). After completing the exposure task, the participants turned their back to the mirror and did not examine their body in the mirror for two minutes. Affect was then recorded for the fourth time (post). Afterwards, the participants rated their state body satisfaction. The follow-up online questionnaire including the assessment of post-event processing had to be answered 24 hours after the exposure (+/- 3 hours; Figure 1). One week later, the procedure was repeated in the same way and by the same examiner. Students received credit points for participation.

### Statistical Analyses

To answer Hypotheses I-III, we conducted mixed analyses of variances (mixed ANOVAs) with two within-subject factors. The first factor *time* had two to four steps depending on the variable: pre-exposure (pre), full-body view at the beginning of exposure (ExB), full-body view at the end of exposure (ExE), and post-exposure (post). The second factor *session* had two steps: first exposure session (S1) and second exposure session (S2). The between-subject factor BDD-*group* was divided into two categories: BDD+ and BDD-. If the assumption of sphericity was not met, we used Greenhouse-Geisser correction. In case of significant effects, post-hoc tests using Bonferroni correction were conducted. To investigate Hypothesis IV, univariate linear regressions were performed. In subsequent sensitivity analyses, the mixed ANOVAs were repeated with a different group factor, categorizing the individuals according to their self-rated BDD diagnosis (DSM-5).

We calculated the sample size with G*Power 3.1 (Faul et al., 2007). For the mixed ANOVAs, we assumed a medium to large effect size for the *group* factor (Kollei & Martin, 2014; Schoenenberg & Martin, 2022; parameters: *f* = 0.35, *r* = 0.50, 1-β = 0.80, α = 0.05) resulting in a sample size of *n* = 44. For the *time* and *session* factors a small to medium effect was expected (Schoenenberg & Martin, 2022; parameters: *f* = 0.17, *r* = 0.70, 1-β = 0.80, α = 0.05) resulting in a sample size of *n* = 44. For the prediction model a medium effect was assumed (parameters: *f*^2^ = 0.15, *r* = 0.70, 1-β = 0.80, α = 0.05) resulting in a sample size of *n* = 43.

Fifty-five people participated in the first session. Five participants (3 BDD+, 2 BDD-), did not participate in the second session and were thus excluded from the sample. One participant (BDD-) reported circulation problems towards the end of the second session and therefore did not provide affect ratings for the measurement of full-body end view (ExE) and post-exposure (Post). Her ratings were constant for the pre-exposure and full-body beginning assessment and were applied for the other two measurements. Hence, a sample of *N* = 50 participants was available for the analysis. The analyses of the PEP-Q after the second session included 45 participants, because five individuals did not respond to this last assessment (3 BDD-, 2 BDD+ cases).

The protocol initially included the assessment of psychophysiological data. Due to severe movement artefacts in the data, we were not able to analyze it.

## Results

### Sample Characteristics

The group with BDD+ did not differ from the BDD- group with regard to age (Table 1), highest education degree, χ^2^(1) = 0.01, or relationship status, χ^2^(2) = 1.02. The mean appearance distortion in the BDD+ group was significantly higher than in the BDD- group. The BDD+ group rated their appearance impairment on average 3.43 points, *SD* = 2.33, lower than the examiners in comparison to the BDD- group, *M* = 1.33, *SD* = 1.52. Individuals with higher BDD symptoms reported higher depressiveness, and a lower BMI (Table 1). All 11 cases that fulfilled the self-rated BDD diagnosis were in the in the BDD+ group.

### Manipulation Check

Both groups were able to follow the mirror exposure instructions to the same extent and were equally motivated to follow them in the first and second sessions (Table 1). Ratings of stress induced by the exposure were significantly higher in the BDD+ group for both sessions (Table 1). Stress induced by exposure dropped from the first to the second exposure by approximately 1.7 points in the BDD+ group and by approximately only 0.5 in the BDD- group.

**Table 1:** Sample Characteristics

Variable	BDD+	BDD-		Total Sample
	*M* (*SD*)	*M* (*SD*)	*t*(*df*)	*M* (*SD*)
**Age**	22.30 (2.82)	23.19 (5.55)	0.69 (48)	22.78 (4.49)
**BMI**	20.81 (1.84)	22.81 (3.41)	2.51 (48)*	21.89 (2.95)
**DCQ Sum Score**	12.35 (2.93)	4.63 (1.84)	-10.92 (35.86)**	8.18 (4.55)
**Appearance Distortion**	3.43 (2.33)	1.33 (1.52)	-3.83 (48)**	2.30 (2.19)
**PHQ-9 Sum Score**	11.17 (4.42)	6.52 (3.81)	-4.00 (48)**	8.66 (4.68)
Ability to Follow Instruction				
First exposure	9.00 (1.78)	9.48 (0.94)	1.22 (48)	9.26 (1.40)
Second exposure	9.00 (1.65)	9.26 (1.20)	0.64 (48)	9.14 (1.41)
Motivation to Follow Instructions				
First exposure	8.04 (1.99)	7.89 (1.40)	-0.32 (48)	7.96 (1.68)
Second exposure	8.09 (1.73)	7.85 (1.41)	-0.53 (48)	7.96 (1.55)
Stress Induced by Exposure				
First exposure	5.26 (2.93)	2.63 (2.31)	-3.36 (48)**	3.84 (2.90)
Second exposure	3.57 (2.19)	2.11 (1.89)	-2.52 (48)**	2.78 (2.14)
	***n***	***n***	**χ^2^**	***n* (%)**
Education			χ^2^(1) = 0.01	
A-levels	22	19		41
University Degree	5	4		9
Relationship Status			χ^2^(2) = 1.02	
Single	11	11		22
Relationship	15	10		25
Married	1	2		3

*Note. N* = 50; *n*_BDD+_ = 23; *n*_BDD-_ = 27; BMI = Body Mass Index; BDD = Body Dysmorphic Disorder; BDD+ = with BDD symptoms; BDD- = without BDD symptoms.

*p < .05. **p < .01.

### Hypotheses

#### BDD Group Effect

Differences between the BDD groups were examined in all analysis using the main effects of group, and time-by-group and session-by-group interaction effects (Table 2). People with distinct BDD symptoms (BDD+ group) showed a significantly lower BISS score, as well as higher levels of shame and sadness during the exposure sessions (Table 3). The session-by-group interaction effect was significant for sadness (Table 2). Post-hoc tests indicated significantly higher sadness for the BDD+ than the BDD- group in the first session but not in the second one (Table 3). Reported tension and anxiety did not differ significantly between the groups. In addition, the degree of post-event processing was significantly higher in the BDD+ than in the BDD- group.

**Table 2:** Results for Repeated Measures Analysis of Variances

Variable/Factor	*F*	*df*	*p*	$\eta_{p}^{2}$
BISS				
Group	6.43	1, 48	.015	.12
Time	14.10	1, 48	< .001	.23
Session	6.02	1, 48	.018	.11
Time x Group	2.15	1, 48	.149	.04
Session x Group	0.19	1, 48	.668	.01
Time x Session	0.10	1, 48	.754	.01
Session x Time x Group	2.34	1, 48	.132	.05
Tension				
Group	1.08	1, 48	.305	.02
Time	14.94	1.93, 92.74	< .001	.24
Session	34.90	1, 48	< .001	.42
Time x Group	1.20	1.93, 92.74	.304	.02
Session x Group	3.57	1, 48	.065	.07
Time x Session	3.11	2.11, 101. 24	.046	.06
Session x Time x Group	0.02	2.11, 101. 24	.982	.00
Shame				
Group	4.67	1, 48	.036	.09
Time	9.24	2.36, 113.45	< .001	.16
Session	29.52	1, 48	< .001	.38
Time x Group	2.17	2.36, 113.45	.110	.04
Session x Group	2.54	1, 48	.118	.05
Time x Session	3.71	1.82, 87.30	.032	.07
Session x Time x Group	0.31	1.82, 87.30	.716	.01
Sadness				
Group	4.52	1, 48	.039	.09
Time	4.93	2.20, 105.69	.007	.09
Session	16.01	1, 48	< .001	.25
Time x Group	1.45	2.20, 105.69	.239	.03
Session x Group	6.07	1, 48	.017	.11
Time x Session	3.07	3, 144	.030	.06
Session x Time x Group	0.71	3, 144	.550	.02
Anxiety				
Group	0.90	1, 48	.348	.02
Time	4.51	2.27, 108.93	.010	.09
Session	7.34	1, 48	.009	.13
Time x Group	1.24	2.27, 108.93	.296	.03
Session x Group	4.02	1, 48	.051	.08
Time x Session	2.06	2.17, 103.96	.129	.04
Session x Time x Group	1.56	2.17, 103.96	.213	.03
PEP-Q				
Group	16.53	1, 43	< .001	.28
Session	13.67	1, 43	.001	.24
Session x Group	0.06	1, 43	.808	.01

*Note. N* = 50; with BDD diagnosis *n* = 11; BDD = Body Dysmorphic Disorder; BISS = Body Image State Scale; PEP-Q = Post-Event Processing Questionnaire.

**Table 3:** Means and Standard Deviation for Affects and Post-Event Processing

Variable	Session 1		Session 2	
	BDD+	BDD-	BDD+	BDD-
	*M* (*SD*)	*M (SD)*	*M (SD)*	*M (SD)*
Tension				
Pre	35.22 (4.70)	28.63 (3.45)	16.52 (3.06)	18.41 (3.30)
ExB	40.87 (5.37)	29.81 (3.53)	19.78 (3.42)	18.15 (3.16)
ExE	36.87 (5.80)	25.74 (4.03)	17.61 (3.80)	16.63 (3.36)
Post	28.13 (5.33)	19.78 (3.68)	13.26 (3.45)	13.70 (3.16)
Shame				
Pre	33.30 (6.32)	20.37 (4.00)	13.26 (2.91)	9.33 (2.67)
ExB	38.26 (6.88)	21.11 (3.93)	18.57 (3.51)	10.44 (2.70)
ExE	35.87 (6.61)	19.00 (4.25)	21.26 (4.35)	10.74 (2.98)
Post	28.70 (5.96)	15.00 (3.46)	16.04 (3.55)	8.33 (2.85)
Sadness				
Pre	10.43 (3.58)	3.52 (2.32)	2.22 (1.08)	1.85 (1.20)
ExB	17.04 (4.91)	5.37 (2.40)	5.96 (2.41)	1.85 (1.20)
ExE	19.13 (4.71)	6.48 (2.60)	5.87 (2.39)	3.33 (1.62)
Post	13.70 (3.89)	4.07 (1.73)	5.00 (2.09)	2.59 (1.50)
Anxiety				
Pre	14.22 (4.67)	9.07 (3.79)	1.48 (1.56)	5.56 (2.12)
ExB	15.22 (5.02)	4.41 (1.93)	3.78 (1.96)	5.00 (2.07)
ExE	10.22 (3.67)	5.19 (2.58)	3.30 (1.79)	3.07 (1.49)
Post	9.13 (3.75)	3.33 (2.07)	3.09 (1.74)	2.96 (1.49)
PEP-Q				
Post	34.40 (21.37)	16.52 (14.80)	27.39 (19.35)	8.89 (8.07)

*Note. n_BDD-_* = 27; *n_BDD+_* = 23; Pre = pre-exposure; ExB = full-body view beginning; ExE = full-body view end; Post = post-exposure; BDD+ = with BDD symptoms; BDD- = without BDD symptoms; BDD = Body Dysmorphic Disorder.

#### Within-Session Time Effect

**Figure 2:**
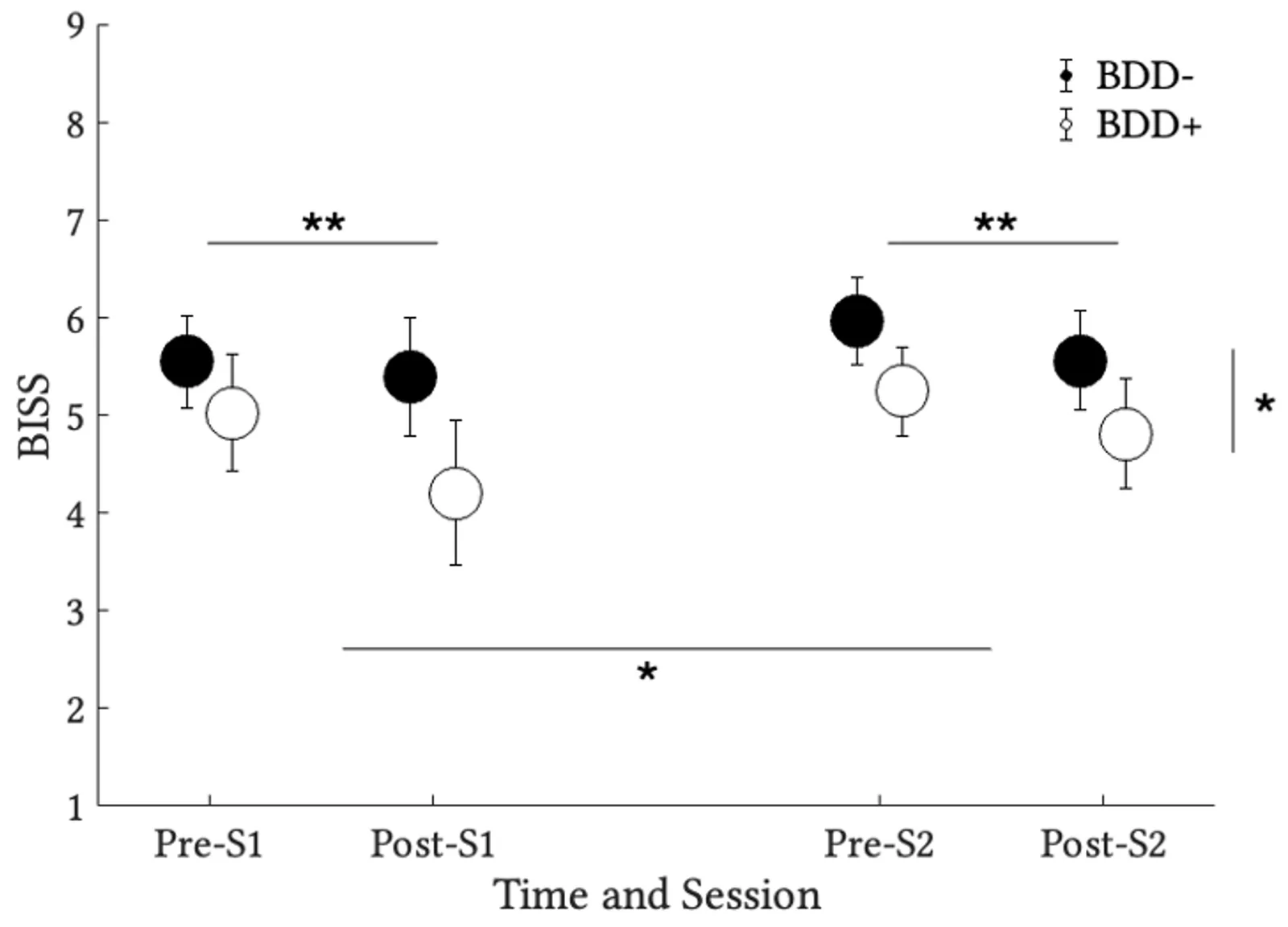
Means and 95% CIs for Body Image Satisfaction *Note. N* = 50; Pre = pre-exposure; Post = post-exposure; S1 = first exposure session; S2 = second exposure session; BISS = Body Image State Scale; BDD+ = with BDD symptoms; BDD- = without BDD symptoms. *p < .05. **p < .01.

The effect of time was examined in all analysis using the main effects of time (Table 2). The BISS score dropped significantly over time within one session (Figure 2). The post-hoc tests confirmed a significant drop in the first, *M*_Diff_ = 0.48, 95% CI [0.13, 0.84], *p* = .008, and in the second sessions, *M*_Diff_ = 0.42, 95% CI [0.14, 0.70], *p* = .004. Overall, the time effects for tension, shame, sadness, and anxiety were significant (Table 2). The post-hoc tests comparing the “full-body view at the beginning” to the “full body view at the end” of each session did not yield significant effects for any affect variable but a significant drops in tension, shame and anxiety “post” the exposure could be detected (Supplementary Materials, Table 2).

#### Between Session Effect

The effect between session was examined in all analysis using the main effects of session (Table 2). The BISS score increased significantly between sessions (Figure 2). Tension, shame, sadness, and anxiety decreased significantly between sessions. The PEP-Q score decreased significantly between sessions (Table 2).

### Model Prediction

The PEP-Q score after the first exposure session was tested as a predictor for the BISS and affect scores at the beginning of the second session. The PEP-Q after the first session positively predicted tension, shame, sadness, and anxiety at the beginning of the “full-body view” in the second session (Table 4). It also significantly predicted shame at the onset of the second session. PEP-Q did not significantly predict BISS prior to the second exposure session.

**Table 4:** Univariate Linear Regression Predicting Body Satisfaction and Affects at the Beginning of the Second Session by Post-Event Processing After the First Session

Predicted Variable	Time 2nd Session	*R* ^2^	*F*	*B*	*SE*	β	*t*	*p*	*B* [95% CI]	
									*LL*	*UL*
**BISS**	Pre	0.04	1.90	-0.01	0.01	-0.20	-1.38	.174	-0.03	0.01
Tension										
	Pre	0.06	3.14	0.20	0.11	0.25	1.77	.083	-0.03	0.42
	ExB	0.25	15.58	0.56	0.14	0.50	3.95**	< .001	0.27	0.84
Shame										
	Pre	0.08	4.20	0.20	0.10	0.28	2.05*	.046	0.01	0.40
	ExB	0.22	13.29	0.65	0.18	0.47	3.65**	.001	0.29	1.01
Sadness										
	Pre	0.01	0.29	0.02	0.04	0.08	0.54	.593	-0.06	0.10
	ExB	0.27	17.53	0.49	0.12	0.52	4.19**	< .001	0.26	0.73
Anxiety										
	Pre	0.01	0.36	0.04	0.07	0.09	0.60	.551	-0.10	0.18
	ExB	0.13	7.31	0.34	0.12	0.36	2.70*	.009	0.09	0.59

*Note. N* = 49; *df* = (1, 48); BISS = Body Image State Scale; Pre = prior to exposure; ExB = full-body view beginning.

*p < .05. **p < .01.

### Sensitivity Analysis

When the groups were categorized according to the self-rated diagnostic criteria, the group main effects were confirmed for the BISS scores, shame, and PEP-Q-scores (Supplementary Materials, Table 3). The main effects for time and session were significant for the BISS score, and all affects; the session effect was significant for the PEP-Q score (Supplementary Materials, Table 3).

## Discussion

The present study was the first to investigate repeated guided mirror exposure in BDD-symptomatic women as a standalone intervention. We aimed to examine effects within one session and between two sessions of mirror exposure.

As assumed, body image exposure was related to a more negative cognitive evaluation, here, state body satisfaction and dysfunctional rumination about the exposure, which is called post-event processing, in individuals with higher BDD symptoms than in those with fewer or no symptoms. Descriptively, both groups experienced tension and shame with the highest intensity, while the levels of anxiety and sadness were overall lower. A comparison of the two groups showed that shame and sadness were elevated only in individuals with distinct BDD symptoms. Sadness was found to be higher for individuals with BDD in the first exposure session only. Unfortunately, we did not capture the level of disgust in this study. Earlier studies have identified the experience of disgust to be relevant during mirror exposure (Kollei & Martin, 2014; Neziroglu et al., 2010).

The results on affects during exposure are in line with the findings of previous studies which found sadness to be higher in individuals with BDD (Kollei & Martin, 2014; Schoenenberg & Martin, 2022) and reported higher levels of shame in BDD positive participants (Schoenenberg & Martin, 2022). Overall, our results indicate that anxiety is not the main affect in this body confrontation paradigm. Shame has been discussed as a key affect in BDD in previous work (Malcolm et al., 2021). Addressing and changing the experience of shame during mirror exposure may be a crucial mechanism that needs further investigation.

The second research question addressed changes over time within one exposure session. State body satisfaction did not increase, but decreased significantly from pre to post in each session. This implies that state body satisfaction deteriorated with the exposure interventions, even though we applied a relatively long exposure time and a neutral description of body parts. Consistent with this, a previous study found increased or unchanged discomfort with one’s body in women who were dissatisfied with their body at the end of a guided mirror exposure (Díaz-Ferrer et al., 2017; Moreno-Domínguez et al., 2012). Affects did not change significantly from the first full-body view at the beginning of the session (ExB) to the full-body view at the end (ExE). It is possible that the participants still felt affected at this point due to the exposure with a difficult stimulus. The intensity of negative affect was significantly lower when exposure was terminated, but negative affects do not seem to decrease during this type of guided exposure. A clear habituation approach without directing attention to the various parts of the body may have led to a greater change in affect from the beginning to the end of the intervention, as it has been found in a study by Díaz-Ferrer et al. (2017) who investigated pure exposure. However, due to the attentional bias associated with body image disorders (Johnson et al., 2018), such an approach would tend to promote a dysfunctional approach to self-image (Veale & Riley, 2001). We did not assess affect while the subjects were viewing their most aversive body part(s). Negative affect might have been increased during this period. In addition, the affect ratings were communicated to the examiner verbally, which may have had an impact on their extend, particularly in the case of shame.

Nevertheless, shame seemed to be more relevant than anxiety during the mirror viewing, as pointed out earlier. The experience of shame is closely related to self-evaluation or evaluation by others (Goss & Allan, 2009). Due to this relationship, changes in shame experience may follow from cognitive changes (e. g. the realization after a mirror exposure that one did not hate the whole body). Consequently, strong affect changes within one session may not be the decisive mechanism for successful mirror exposure and long-term changes in shame experience.

The comparison of the exposure sessions revealed that state body satisfaction was significantly higher and negative affects were significantly lower in the second session, indicating the positive effect of the repeated exposure intervention. An increase of body satisfaction between sessions was also reported in a study of women with bulimic symptoms (Díaz-Ferrer et al., 2015) and later in a study of body dissatisfied and subclinical eating disordered women (Díaz-Ferrer et al., 2017), both studies used pure and guided exposure. In line with our results, Trentowska et al. (2013) reported a reduction of subjective distress and negative affect between sessions using a similar approach to that used in this study, including guiding attention and a neutral description of the various body parts. A study by Tanck et al. (2021) found positive between-session effects among healthy women in terms of body satisfaction and negative affect for both types of instructions – positive or negative verbalizations – of body parts during exposure. Taken together, all of these findings imply the importance of repeated mirror exposure in clinical practice even though the possible different underlying mechanisms have not yet been clarified.

Another positive effect of repeated mirror exposure was the lower level of post-event processing after the second session. Experiencing the second exposure as less aversive could have reduced engagement in ruminative processes afterwards. Furthermore, higher post-event processing after the first exposure predicted higher tension, shame, sadness, and fear when individuals were initially confronted with their body in the mirror at the second exposure. Addressing dysfunctional post-processing and providing more helpful ways to deal with uncertainty could therefore be very important interventions for clinical practice. Due to the potential impact on memory and associated expectations, addressing post-event processing could possibly amplify the positive effects or reduce negative effects due to dysfunctional processing between exposure sessions.

In sum, repeated mirror exposure may improve important processes, and post-event possessing may be an indicator for successful coping or learning. However, this assumption needs further scientific investigation. Mechanisms of mirror exposure for eating disorders have been discussed by Naumann et al. (2022). They proposed cognitive restructuring and related changes in cognitive biases as plausible explanations. In this study, we did not induce any specific expectations regarding the effects of the exposure and did not examine the expectations of participants. Therefore, we cannot accurately assess the role of expectations in this context. Since current research on anxiety disorders point to the important role of changes in expectations during exposure and the exposure-related learning rate (Pittig et al., 2023), it would be very important to investigate the role of expectation changes and learning rate through mirror exposure in the future. We should incorporate the assessment of expectations prior to the intervention and understand how expectation may influence the results.

Some limitations of the present study need to be considered when interpreting the results. One main limitation is the not fully clinical sample and the lack of confirmation of the clinical diagnosis by means of a clinical interview. Individuals with BDD symptoms who strongly avoid mirrors probably did not take part in the study. Differential effects may become apparent in comparison to a fully clinical sample. We investigated women only, even though there is a substantial group of males with BDD. The internal description of appearance during exposure may have reduced the intensity of affect intensity. Although participants said they were able to follow the instructions, we cannot know, i.e., whether they shifted their visual attention to different body parts. In addition, possibly ongoing safety behavior could have been better recognized by a therapist guiding the participant. We focused on assessing in-session and short-term between-session effects. Hence, further investigation of the stability of the improvements is required. It is of interest to see whether further sessions, including repetition as homework, contribute to an even greater change in individuals with BDD.

### Conclusions

In sum, in spite of the limitations, this research provides further insights into mirror exposure in individuals with BDD symptoms. It presents evidence for a positive effect of repeated guided mirror exposure in terms of state body image and affective response in women. The role of cognitive post-processing of mirror confrontation is highlighted, and new research questions are derived to address the key change mechanisms of mirror exposure.

## Supplementary Materials

The Supplementary Materials (Schoenenberg & Martin, 2026S) include a table listing the interview questions about the exposure experience (Supplementary Table 1), post-hoc tests on affect for the factor time (Supplementary Table 2), and results for the Repeated Measures Analysis of Variances comparing the group with BDD-Diagnosis to the group without diagnosis (Supplementary Table 3).



SchoenenbergK.
MartinA.
 (2026S). Supplementary materials to "Repeated mirror exposure in individuals with body dysmorphic symptoms"
[Supplementary tables]. PsychOpen. 10.23668/psycharchives.21860


## Data Availability

The data and materials that support the findings of this study are available from the corresponding author upon reasonable request.

## Preregistration

The study was not submitted to a preregistration platform.

## Reporting Guidelines

The study adheres to the JARS-Quant Guidelines of the American Psychological Association.
